# CTE neuropathology alone is associated with dementia and cognitive symptoms

**DOI:** 10.1002/alz.71032

**Published:** 2026-01-27

**Authors:** Rachael M. Layden, Jenna R. Groh, Annalise E. Miner, Abigail Kidd, Sophia B. Nosek, Stephanie Gonzalez Gil, Bobak Abdolmohammadi, Steven Lenio, Christopher J. Nowinski, Yorghos Tripodis, Brett M. Martin, Joseph N. Palmisano, Brigid C. Dwyer, Douglas I. Katz, Lee E. Goldstein, Robert C. Cantu, Robert A. Stern, Thor D. Stein, Ann C. McKee, Daniel H. Daneshvar, Jesse Mez, Michael L. Alosco

**Affiliations:** ^1^ Boston University Alzheimer's Disease Research Center and Boston University CTE Center Boston University Chobanian & Avedisian School of Medicine Boston Massachusetts USA; ^2^ Department of Neurology Boston University Chobanian & Avedisian School of Medicine Boston Massachusetts USA; ^3^ Department of Neurology Boston Medical Center Boston Massachusetts USA; ^4^ Concussion and CTE Foundation Boston Massachusetts USA; ^5^ Department of Biostatistics Boston University School of Public Health Boston Massachusetts USA; ^6^ Center for Health Data Science Boston University School of Public Health Boston Massachusetts USA; ^7^ Department of Pathology and Laboratory Medicine Boston University Chobanian & Avedisian School of Medicine Boston Massachusetts USA; ^8^ Department of Psychiatry Boston University Chobanian & Avedisian School of Medicine Boston Massachusetts USA; ^9^ Departments of Biomedical Electrical & Computer Engineering Boston University College of Engineering Boston Massachusetts USA; ^10^ Department of Neurosurgery Boston University Chobanian & Avedisian School of Medicine Boston Massachusetts USA; ^11^ Department of Neurosurgery Emerson Hospital Concord Massachusetts USA; ^12^ Department of Anatomy & Neurobiology Boston University Chobanian & Avedisian School of Medicine Boston Massachusetts USA; ^13^ US Department of Veterans Affairs VA Boston Healthcare System Jamaica Plain Massachusetts USA; ^14^ U.S. Department of Veterans Affairs VA Bedford Healthcare System Bedford Massachusetts USA; ^15^ Framingham Heart Study Boston University Chobanian & Avedisian School of Medicine Boston Massachusetts USA; ^16^ Department of Physical Medicine and Rehabilitation Harvard Medical School Boston Massachusetts USA; ^17^ Department of Physical Medicine and Rehabilitation Massachusetts General Hospital Boston Massachusetts USA; ^18^ Department of Physical Medicine and Rehabilitation Mass General Brigham‐Spaulding Rehabilitation Charlestown Massachusetts USA

**Keywords:** Alzheimer's disease, chronic traumatic encephalopathy, concussion, dementia, neurodegenerative disease, repetitive head impacts

## Abstract

**INTRODUCTION:**

This studyexamined the independent contribution of chronic traumatic encephalopathy (CTE) neuropathology to symptoms.

**METHODS:**

The sample included 614 brain donors with (*n* = 366) and without (*n* = 248) autopsy‐confirmed CTE. Brain donors with other major neurodegenerative disease diagnoses were excluded. Informants completed cognitive and neuropsychiatric measures. Dementia was determined during diagnostic consensus conferences.

**RESULTS:**

CTE stage IV (of IV) was associated with 4.48 (95% confidence interval [CI] = 1.97–10.90) increased odds of having dementia. CTE stage III had an odds ratio of 2.12 (95% CI = 1.91–3.77). Higher CTE stage was associated with greater informant‐reported cognitive symptoms (*p* < 0.01). There were no associations with mood/behavioral scales.

**DISCUSSION:**

CTE stage III/IV neuropathology was associated with dementia and cognitive symptoms: those with stage IV were 4.5 times more likely to have dementia than those without CTE. It is uncertain if low‐stage CTE clinically manifests, and mood/behavioral symptoms likely have multifactorial causes and/or a fluctuating course.

**Highlights:**

- Stage III and IV chronic traumatic encephalopathy (CTE) are independently associated with increased odds of having dementia.
- Higher CTE stage was associated with greater informant‐reported cognitive symptoms.
- Stage I and II CTE were not associated with cognitive symptoms or dementia.
- CTE of any severity was not associated with informant‐reported mood or behavioral symptoms.

## 1 INTRODUCTION

Chronic traumatic encephalopathy (CTE) has been neuropathologically diagnosed in people exposed to repetitive head impacts (RHI) from contact and collision sport play, including American football and others.^1^, ^2^, ^3^, ^4^ The unique diagnostic lesion of CTE is hyperphosphorylated tau (p‐tau) protein in neurons around small blood vessels at the depths of the sulci.^5^, ^6^ In stage I CTE, there are one to two epicenters of p‐tau, typically in the frontal cortices. Stage II CTE involves multiple foci of p‐tau in the cortex, spreading to the temporal and parietal lobes. By stage III, there is involvement of medial temporal lobe structures. Stage IV CTE is characterized by widespread p‐tau pathology across cortical and subcortical structures.^5^, ^6^

CTE neuropathology cannot accurately be detected during life at this time. Research diagnostic criteria for the clinical syndrome of CTE are known as traumatic encephalopathy syndrome or TES.^7^ To meet TES criteria, there must be substantial exposure to RHI and the presence of a progressive core clinical feature (i.e., cognitive impairment and/or neurobehavioral dysregulation) that is not better accounted for by another condition or disorder. Levels of certainty of underlying CTE neuropathology (suggestive, possible, probable) can be determined, which are in part informed by dementia status and the presence of supportive features.^7^ The TES criteria were developed to reflect the clinical symptoms typically associated with CTE p‐tau pathology, but the neuropathological correlates of some of these symptoms are unknown. Particularly in older age and advanced stages, CTE is highly co‐morbid with other neuropathologies (e.g., Alzheimer's disease [AD], Lewy body disease [LBD], white matter and vascular injury, TAR DNA‐binding protein 43 [TDP‐43]), and exposure to RHI has been associated with mixed neuropathologies independent of CTE.^8^, ^9^, ^10^, ^11^, ^12^ People at high risk for CTE also have a wide range of medical, health, and psychiatric co‐morbidities that can affect cognitive and neuropsychiatric function.^13^, ^14^ Consequently, there has been uncertainty about the independent contribution of CTE neuropathology to clinical symptoms.^10^ This is especially true for low‐stage CTE (stages I and II), and whether there is sufficient tau pathology at this stage of the disease to result in symptoms.

A recent review and meta‐analysis revealed cognitive impairments and dementia to be common in autopsy‐confirmed CTE; however, the authors concluded the contribution of CTE to symptoms was uncertain given the high rates of co‐morbid neuropathologies.^10^ In a sample of 366 male brain donors neuropathologically diagnosed with CTE, Alosco et al. examined the relationship between the McKee CTE staging scheme and dementia. Higher CTE stage was associated with increased odds of having *ante mortem* dementia.^15^ In another study of 364 brain donors with autopsy‐confirmed CTE, global, frontal, inferior parietal, superior temporal, and amygdala CTE p‐tau severity was associated with worse scores on informant‐reported cognitive and functional scales. While CTE p‐tau severity was associated with one scale of neurobehavioral dysregulation, the effect size was weak.^16^ These initial studies on CTE and symptoms did not fully isolate the effects of CTE neuropathology alone or examine the minimum CTE neuropathology thresholds necessary to cause symptoms. In a case series of 152 brain donors younger than 30 years old, there was no association between CTE status and informant‐reported clinical symptoms.^17^ Most donors in that sample who had CTE had low‐stage CTE (60/63, 95.2%), creating uncertainty about the contribution of low‐stage CTE pathology alone to symptoms. However, a recent study identified multiple non‐tau pathologies, including neuroinflammation and neuronal loss, at the sulcal depths in young individuals with RHI exposure (< 52 years old), even in the absence of CTE lesions. The contribution of these pathologies to symptoms among individuals with and without CTE is uncertain.^18^

In the largest study of its kind, we examined the association between CTE stage and cognitive, functional, and neuropsychiatric symptoms among 614 brain donors with (*n* = 366) and without (*n* = 248) CTE. We tested associations across each CTE stage to increase understanding of the threshold of p‐tau pathology needed to confer symptom manifestation. All associations were tested after exclusion of AD, LBD, frontotemporal lobar degeneration (FTLD), and motor neuron disease (MND). We statistically accounted for vascular pathologies, Braak staging of neurofibrillary tangles, hippocampal sclerosis, and substance use treatment history. In so doing, the current study provides unique insights into the cognitive, functional, and neuropsychiatric correlates of CTE neuropathology.

**RESEARCH IN CONTEXT**

1. **Systematic review**: We reviewed the literature using PubMed and the references of research articles. Previous studies have shown associations between chronic traumatic encephalopathy (CTE) neuropathology and symptoms. However, the literature is mixed, and studies that have found associations included samples with co‐morbid neuropathologies and did not examine the threshold of CTE neuropathology necessary to confer symptoms. We examined the association between CTE stage and cognitive, functional, and neuropsychiatric symptoms among 614 brain donors with (*n* = 366) and without (*n* = 248) CTE. We tested associations across each CTE stage to increase understanding of the threshold of phosphorylated tau pathology needed to confer symptom manifestation.
2. **Interpretation**: CTE stage III/IV neuropathology is associated with dementia and cognitive symptoms in the absence of co‐morbid neurodegenerative disease. It is uncertain if low‐stage CTE is sufficient to clinically manifest, and mood/behavioral symptoms likely have multifactorial causes and/or a fluctuating course.
3. **Future directions**: Prospective clinical–pathological studies will be needed to validate the associations between CTE neuropathology and objectively measured symptom phenotypes.

## 2 METHODS

### 2.1 Participants

The original sample included 901 brain donors with RHI exposure from the Understanding Neurologic Injury and Traumatic Encephalopathy (UNITE) brain bank. The details of the UNITE study have been described previously.^11^, ^19^ The next of kin contacted the UNITE brain bank near the time of death for most donations, with some donors referred by the Concussion and CTE Foundation or medical examiners, and some who expressed a desire to donate their brain after death via the UNITE Brain Donation Registry. The donor's next of kin or legally authorized representative provided consent for the donation of brain tissue. The inclusion criteria for the UNITE study do not require the presence of symptoms. RHI exposure criteria include those who played contact sports at any level, were exposed to military combat exposure, had a history of intimate partner violence, or other sources. The criteria exclude specimens for a prolonged *post mortem* interval, and insufficient and/or poor specimen quality that precluded neuropathological diagnosis. The institutional review board at Boston University Medical Center approved all brain donation procedures, including *post mortem* clinical record review, interviews with informants, and neuropathological evaluation.

### 2.2 Sample selection

**FIGURE 1:**
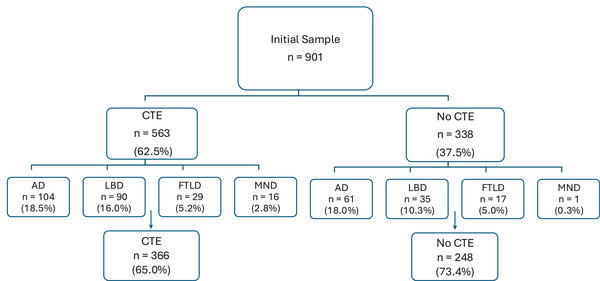
Sample selection. The flow chart illustrates the exclusions for neurodegenerative disease diagnoses. Donors with AD, LBD, FTLD, and MND were excluded. The number of co‐morbid diagnoses exceeds the number of excluded cases, as several donors were diagnosed with more than one pathology. Additionally, one donor was excluded from the CTE group due to a diagnosis of alcohol‐related neurodegeneration consistent with Wernicke's encephalopathy, and two donors were excluded from the no CTE group due to missing AD diagnostic information. In addition, sample sizes varied across outcomes due to missingness. AD, Alzheimer's disease; CTE, chronic traumatic encephalopathy; FTLD, frontotemporal lobar degeneration; LBD, Lewy body disease; MND, motor neuron disease

Figure 1 provides an overview of sample selection. Of the 901 brain donors, 563 had CTE. All brain donors with the following non‐CTE neurodegenerative disease diagnoses were excluded for the purposes of this study, including AD (intermediate to high based on National Institute on Aging [NIA]‐Reagan), LBD (limbic or neocortical), MND, and FTLD. One case was removed due to alcohol‐related neurodegeneration consistent with Wernicke's encephalopathy. This excluded 196 cases. This resulted in a final analytic sample of 614 brain donors, including 366 donors with and 248 without autopsy‐confirmed CTE neuropathology. Sample sizes also varied across outcomes due to missing data. Co‐pathology exclusion was not exhaustive, and sensitivity models accounted for other potential pathologies that can contribute to cognitive, mood, and behavioral symptoms. For example, we did not exclude cerebrovascular neuropathologies due to their high frequency in the sample. Instead, these neuropathologies were included as covariates in statistical models.

### 2.3 Informant retrospective clinical evaluation: dementia diagnosis

Informants of the donors completed retrospective clinical evaluations that combined online surveys and telephone interviews with medical records. Information was collected on donor demographics; education attainment; athletic history (e.g., sports played, level of play, position, age of first exposure, and duration); military history; traumatic brain injury history; substance use; and medical, social, and family histories of the donors. The clinical interviews were performed by a neurologist, behavioral neuroscientist, neuropsychologist, or trained research staff. Clinicians and informants were blinded to neuropathological diagnoses at the time of the informant retrospective interviews. The clinical symptoms and disease course progression were described in a report reflecting information gathered during the informant interview. Dementia was determined by diagnostic consensus conferences that included a panel of expert clinicians. Dementia diagnoses were based on the Diagnostic and Statistical Manual of Mental Disorders Fourth Edition Text Revision criteria.^20^

### 2.4 Informant retrospective clinical evaluation: clinical scales

Informants completed modified scales assessing the donor's cognition, function, mood, and behavior. Detailed information on the scales and their scoring is reported elsewhere.^16^ Cognitive symptoms were measured by the Cognitive Difficulties Scale (CDS) and the Behavior Rating Inventory of Executive Function‐A (BRIEF‐A) Metacognition Index (MI). The Functional Activities Questionnaire (FAQ) assessed instrumental activities of daily living. Mood measures included the Geriatric Depression Scale‐15 (GDS‐15), Beck Anxiety Inventory (BAI), and Apathy Evaluation Scale (AES). Neurobehavioral dysregulation measures included the Barratt Impulsiveness Scale‐11 (BIS‐11) and BRIEF‐A Behavior Regulation Index (BRI). For all scales, higher scores are indicative of greater symptom severity.

### 2.5 Neuropathological diagnoses

Neuropathologists made CTE and CTE staging diagnoses, blinded to RHI exposure history and clinical data during the examination of the brain tissue. The methodological procedures for neuropathological processing and evaluation have been described elsewhere.^2^, ^15^, ^16^, ^21^ The neuropathological diagnosis of CTE was made using neuropathological criteria defined by an National Institute of Neurological Disorders and Stroke/National Institute of Biomedical Imaging and Bioengineering consensus panel.^5^, ^6^ These CTE criteria require perivascular p‐tau in neurons, with or without astrocytes, at the depths of a cortical sulcus.^6^ CTE severity was graded using the four‐stage McKee CTE classification scheme based on the extent and severity of p‐tau pathology.^15^, ^22^ Ratings for the CTE stage have been shown to have good interrater reliability.^15^ Discrepancies between neuropathological diagnoses were resolved by discussion and consensus among neuropathologists.^15^ The primary independent variable was the ordinal ranked variable CTE stage (0–IV). Other neuropathologies included in this study were arteriolosclerosis, atherosclerosis, white matter rarefaction, hippocampal TDP‐43 inclusions, and hippocampal sclerosis. Methods for the evaluation and diagnoses of these have been provided elsewhere.^11^, ^23^, ^24^

### 2.6 Statistical analyses

All analyses were performed using SPSS (IBM SPSS Statistics, Version 29.0.20) and RStudio (Version 4.4.0, 2024.04.24). *t* tests (or Wilcoxon rank sum test when non‐normally distributed) and Pearson chi‐squared tests (or Fisher exact tests) were used to compare sample characteristics by CTE stage. Binary logistic regressions tested the association between CTE stage and dementia status (yes/no) with no CTE as the reference group. Analyses of covariance compared cognitive, mood, and behavioral scales (i.e., CDS, BRIEF‐A MI, FAQ, GDS‐15, BAI, AES, BIS‐11, and BRIEF‐A BRI) across CTE stages (none, I, II, III, IV). A separate model was performed for each scale. When a significant main effect of CTE stage was presented, pairwise post hoc comparisons were performed using Tukey's honestly significant difference adjustment for multiple testing. Statistical significance was set at *p* < 0.05 for all analyses. Because there were only two analyses per domain (i.e., cognition, dementia, mood, and neurobehavioral dysregulation), adjustment for multiple comparisons was not performed.

All models controlled for age at death. Models were repeated with arteriolosclerosis, atherosclerosis, white matter rarefaction, and substance use treatment history included as covariates. Additional sensitivity analyses were conducted with Braak stage and hippocampal sclerosis as covariates to the main model. Cerebrovascular pathologies were coded as none/mild and moderate/severe, and substance use treatment history was coded as binary (yes/no). Hippocampal sclerosis was similarly coded as binary (present/absent), and Braak stage was coded as 0 to VI.

Consistent with recent recommendations for more practical and reliable staging of CTE, primary models were repeated with CTE stages I and II categorized as “low” and stages III and IV categorized as “high,” independently compared to the no CTE group.^6^ The primary models were also repeated stratified by the median age of the sample (52 years) to attenuate concerns related to age differences by CTE stage in the entire sample.

## 3 RESULTS

### 3.1 Sample characteristics

Sample clinical and demographic data by CTE stage are presented in Table 1. Of the sample, 366 (59.60%) had CTE, and 248 (40.19%) did not. Five hundred ninety‐four (97%) were male, and 20 (3.3%) were female. Ninety‐eight (15.96%) were Black/African American, and 500 (81.43%) were White. The mean age of death was 51.99 (standard deviation [SD] = 20) with ages ranging from 13 to 98. Those who had CTE stages III and IV were more likely to be male and older compared to no CTE and CTE stages I and II (*p* < 0.0001). The sample predominantly included brain donors who played American football (*n* = 493, 80.3%), with the highest level of play being youth for 23 donors (4.7%), 106 high school (22%), 152 college (31%), 21 semi‐professional (4.3%), and 188 (38%) played at the professional level. Those with more severe CTE had more years of American football play (*p* < 0.001) and played at a higher level (*p* < 0.001).

**TABLE 1:** Sample characteristics by CTE stage.

	*N*	Stage 0 (*N* = 248^a^)	Stage I (*N* = 89^a^)	Stage II (*N* = 82^a^)	Stage III (*N* = 135^a^)	Stage IV (*N* = 60^a^)	*p* value^b^
Age of death, mean (SD), [range]	614	44.3 (19.0) [13.0, 97.0]	42.3 (16.1) [21.0, 82.0]	48.4 (16.7) [18.0, 88.0]	64.7 (15.2) [29.0, 96.0]	74.6 (9.1) [42.0, 98.0]	<0.001
Sex (% male)	614	229 (92%)	88 (99%)	82 (100%)	135 (100%)	60 (100%)	<0.001
Race	604						<0.001
White		225 (93%)	67 (77%)	64 (80%)	92 (69%)	52 (87%)	
Black		16 (6.6%)	19 (22%)	14 (18%)	41 (31%)	8 (13%)	
Other		2 (0.8%)	1 (1.1%)	2 (2.5%)	1 (0.7%)	0 (0%)	
Education level	614						
Some high school or less		20 (8.1%)	1 (1.1%)	1 (1.2%)	0 (0%)	0 (0%)	
High school diploma/GED		40 (16%)	4 (4.5%)	1 (1.2%)	4 (3.0%)	2 (3.3%)	
Some college		60 (24%)	21 (24%)	22 (27%)	22 (16%)	7 (12%)	
College degree or higher		128 (52%)	63 (71%)	58 (71%)	109 (81%)	51 (85%)	
Years of football play	493	8.3 (5.0)	11.4 (4.5)	13.4 (5.1)	14.8 (5.3)	16.5 (5.6)	<0.001
Highest level of football play	490						<0.001
Pre high school		16 (10%)	4 (5.4%)	3 (4.2%)	0 (0%)	0 (0%)	
High school		77 (48%)	15 (20%)	6 (8.5%)	5 (3.9%)	3 (5.3%)	
College		46 (29%)	26 (35%)	28 (39%)	40 (31%)	12 (21%)	
Semi‐professional		8 (5.0%)	6 (8.1%)	2 (2.8%)	4 (3.1%)	1 (1.8%)	
Professional		13 (8.1%)	23 (31%)	32 (45%)	79 (62%)	41 (72%)	
Position played at highest level	467						<0.001
Offensive line		25 (17%)	13 (18%)	13 (19%)	24 (19%)	9 (16%)	
Tight end		1 (0.7%)	1 (1.4%)	2 (2.9%)	8 (6.3%)	2 (3.5%)	
Quarterback		8 (5.6%)	6 (8.3%)	3 (4.4%)	2 (1.6%)	3 (5.3%)	
Running back		11 (7.7%)	3 (4.2%)	5 (7.4%)	21 (17%)	12 (21%)	
Wide receiver		4 (2.8%)	3 (4.2%)	4 (5.9%)	5 (3.9%)	2 (3.5%)	
Defensive line		14 (9.8%)	16 (22%)	12 (18%)	18 (14%)	6 (11%)	
Linebacker		11 (7.7%)	4 (5.6%)	16 (24%)	20 (16%)	10 (18%)	
Defensive back		10 (7.0%)	10 (14%)	5 (7.4%)	21 (17%)	6 (11%)	
Other/special teams		6 (4.2%)	3 (4.2%)	3 (4.4%)	0 (0%)	0 (0%)	
Multiple		53 (37%)	13 (18%)	5 (7.4%)	8 (6.3%)	7 (12%)	
Played other contact sports	614	136 (55%)	45 (51%)	39 (48%)	41 (30%)	16 (27%)	<0.001
Treated for drugs or alcohol	565	81 (36%)	28 (35%)	32 (43%)	33 (26%)	13 (24%)	0.049
Cause of death	611						<0.001
Suicide		98 (40%)	31 (35%)	21 (26%)	18 (13%)	2 (3.4%)	
Accidental overdose		29 (12%)	11 (12%)	13 (16%)	3 (2.2%)	1 (1.7%)	
Cardiovascular disease		39 (16%)	12 (13%)	17 (21%)	32 (24%)	4 (6.8%)	
Neurodegenerative		8 (3.2%)	1 (1.1%)	3 (3.7%)	24 (18%)	33 (56%)	
Cancer		16 (6.5%)	3 (3.4%)	5 (6.2%)	16 (12%)	5 (8.5%)	
Other		42 (17%)	19 (21%)	14 (17%)	40 (30%)	13 (22%)	
Injury		15 (6.1%)	12 (13%)	8 (9.9%)	2 (1.5%)	1 (1.7%)	

Abbreviations: CTE, chronic traumatic encephalopathy; GED, General Educational Development test; SD, standard deviation.

^a^ Mean (SD); *n* (%). If missingness, sample sizes may vary by CTE stage.

^b^ Kruskal–Wallis rank sum test; Fisher exact test; Pearson chi‐squared test; NA.

Neuropathological characteristics are presented in Table 2. Two hundred fifty (40.72%) had neuropathological diagnoses of arteriolosclerosis, 66 (10.75%) had atherosclerosis, 228 (37.13%) had white matter rarefaction, and 67 (10.91%) had hippocampal sclerosis. Hippocampal TDP‐43 inclusions were present and not excluded or controlled for because nearly all of those with inclusions had CTE stage III or IV (76/83, 91.6%) and dementia (70/83, 84.3%). The cell sizes of those without TDP‐43 inclusions and without dementia are insufficient for statistical analyses. Exclusion for this variable would result in the removal of 66 cases who had stage III and IV and dementia.

**TABLE 2:** Neuropathological characteristics by CTE stage.

	*N*	CTE stage 0 *N* = 248^a^	CTE stage I *N* = 89^a^	CTE stage II *N* = 82^a^	CTE stage III *N* = 135^a^	CTE stage IV *N* = 60^a^	*p* value^b^
Arteriosclerosis	614						<0.001
None/mild		173 (70%)	66 (74%)	60 (73%)	53 (39%)	12 (20%)	
Moderate/severe		75 (30%)	23 (26%)	22 (27%)	82 (61%)	48 (80%)	
Atherosclerosis	565						<0.001
None/mild		211 (95%)	78 (96%)	71 (90%)	101 (81%)	38 (66%)	
Moderate/severe		11 (5.0%)	3 (3.7%)	8 (10%)	24 (19%)	20 (34%)	
White matter rarefaction	612						<0.001
None/mild		175 (71%)	67 (75%)	66 (80%)	65 (49%)	11 (18%)	
Moderate/severe		72 (29%)	22 (25%)	16 (20%)	69 (51%)	49 (82%)	
Braak stage	613						–
Stage 0/B1		196 (79%)	61 (69%)	31 (38%)	7 (5.2%)	0 (0%)	
Stage I/B1		17 (6.9%)	13 (15%)	24 (29%)	6 (4.4%)	0 (0%)	
Stage II/B1		14 (5.7%)	9 (10%)	22 (27%)	36 (27%)	8 (13%)	
Stage III/B2		11 (4.5%)	4 (4.5%)	2 (2.4%)	61 (45%)	31 (52%)	
Stage IV/B2		9 (3.6%)	1 (1.1%)	2 (2.4%)	19 (14%)	20 (33%)	
Stage V/B3		0 (0%)	0 (0%)	0 (0%)	4 (3.0%)	1 (1.7%)	
Stage VI/B3		0 (0%)	0 (0%)	0 (0%)	1 (0.7%)	0 (0%)	
Unable to be determined		0 (0%)	1 (1.1%)	1 (1.2%)	1 (0.7%)	0 (0%)	
CERAD score^*^	614						–
No neuritic plaques		236 (95%)	88 (99%)	80 (98%)	111 (82%)	33 (55%)	
Sparse neuritic plaques		12 (4.8%)	1 (1.1%)	2 (2.4%)	24 (18%)	27 (45%)	
Moderate neuritic plaques		0 (0%)	0 (0%)	0 (0%)	0 (0%)	0 (0%)	
Frequent neuritic plaques		0 (0%)	0 (0%)	0 (0%)	0 (0%)	0 (0%)	
Thal phase	614						–
Phase 0		192 (77%)	75 (84%)	64 (78%)	75 (56%)	13 (22%)	
Phase 1		24 (9.7%)	5 (5.6%)	12 (15%)	17 (13%)	8 (13%)	
Phase 2		7 (2.8%)	2 (2.2%)	1 (1.2%)	13 (9.6%)	4 (6.7%)	
Phase 3		23 (9.3%)	7 (7.9%)	4 (4.9%)	16 (12%)	10 (17%)	
Phase 4		1 (0.4%)	0 (0%)	1 (1.2%)	10 (7.4%)	20 (33%)	
Phase 5		1 (0.4%)	0 (0%)	0 (0%)	4 (3.0%)	5 (8.3%)	
Hippocampal TDP‐43	605	2(0.8%)	2 (2.3%)	3 (3.7%)	36 (27%)	40 (67%)	<0.001
Hippocampal sclerosis	599	3(1.3%)	2 (2.4%)	3 (3.7%)	29 (21%)	30 (50%)	<0.001

Abbreviations: CERAD, Consortium to Establish a Registry for Alzheimer's Disease; CTE, chronic traumatic encephalopathy; TDP‐43, TAR DNA‐binding protein 43.

^a^ *n*(%). If missingness, sample sizes may vary by CTE stage.

^b^ Pearson chi‐squared test;

* Indicates variables or groups for which comparisons were not made due to insufficient cell counts.

As shown in Table 3, of brain donors with autopsy‐confirmed stage III or IV (*n* = 195), dementia was diagnosed by a physician during life in 99 (50.8%). The most common suspected etiological diagnosis was AD (40%), followed by unspecified or unknown (38%). CTE (17%) and vascular dementia (10%) then followed. Only 12 of the 171 brain donors with CTE stage I and II at autopsy were diagnosed with dementia by a physician during life.

**TABLE 3:** Neurological diagnoses made during life of brain donors with autopsy‐confirmed CTE stage III and IV (*N* = 195).

Suspected type or cause of dementia (by physician)	Dementia (diagnosed during life by physician) *N* = 99
Alzheimer's disease	40 (40%)
Dementia unspecified or unknown	38 (38%)
Chronic traumatic encephalopathy	17 (17%)
Vascular dementia	10 (10%)
Frontotemporal dementia^a^	8 (8.1%)
Lewy bodies/Parkinson's disease dementia	6 (6.1%)
Alcohol related dementia	3 (3.0%)
Dementia due to traumatic brain injury	1 (1.0%)

These are all diagnoses made by a physician during life and not those made by researchers as part of the UNITE study. More than one etiology is possible.

^a^ Five cases were behavioral variant, and one was primary progressive aphasia. Two cases with FTD were suspected, but unclear if officially diagnosed and/or if they were behavioral variant or primary progressive aphasia.

Abbreviations: CTE, chronic traumatic encephalopathy; FTD, frontotemporal dementia; UNITE, Understanding Neurologic Injury and Traumatic Encephalopathy.

### 3.2 CTE stage and dementia

Of the sample, 186 (31%) had a dementia diagnosis as made by the panel of clinicians from the UNITE study. Of those with dementia (*n* = 186), 39 (21%) had no CTE, 11 (6%) had stage I, 19 (10%) had stage II, 70 (38%) had stage III, and 47 (25%) had stage IV. Of the 20 females in the sample, one had stage I CTE without dementia. Three females had a diagnosis of dementia without CTE but had mild to moderate arteriosclerosis and white matter rarefaction. Of the 39 donors (16%) who had dementia and no CTE, 4 (10.3%) had neuropathological diagnoses of moderate to severe atherosclerosis, 25 (64.1%) had arteriosclerosis, and 21 (53.8%) had white matter rarefaction.

Table 4 shows the associations among CTE stage, dementia status, and the cognitive scales. When accounting for age of death, CTE stage IV was associated with 4.48 (95% confidence interval [CI] = 1.97–10.90, *p* = 0.001) increased odds of having dementia compared to no CTE. CTE stage III was associated with 2.12 times increased odds of having dementia compared to no CTE (95% CI = 1.91–3.77, *p* = 0.011). CTE stages I and II were not associated with increased odds of having dementia compared to no CTE. When white matter rarefaction and cerebrovascular pathologies and substance use treatment were included in the model, CTE stage IV was associated with a 4.13 times increased odds for dementia (95% CI = 1.71–10.74, *p* = 0.002). The effect for CTE stage III diminished (odds ratio [OR] = 1.83, 95% CI = 0.99–3.39, *p* = 0.055). Results were similar when Braak stage was included in the model: CTE stage IV was associated with 3.88 increased odds for dementia (95% CI = 1.52–10.48, *p* = 0.006), and the effects for CTE stage III were diminished (OR = 1.86, 95% CI = 0.91–3.80, *p* = 0.087). Results when hippocampal sclerosis was added to the model included: CTE stage IV was associated with 2.80 increased odds for dementia (95% CI = 1.17–7.11, *p* = 0.024), and the effect for CTE stage III was diminished (OR = 1.76, 95% CI = 0.97–3.19, *p* = 0.064).

**TABLE 4:** Cognitive, functional, and neuropsychiatric scale summary statistics by CTE stage.

	*N*	CTE stage 0 *N* = 248^a^	CTE stage I *N* = 89^a^	CTE stage II *N* = 82^a^	CTE stage III *N* = 135^a^	CTE stage IV *N* = 60^a^	*p* value
Cognitive Difficulties Scale total score	613	62.3 (38.4)	56.7 (35.3)	63.0 (37.2)	84.5 (43.4)	116.4 (37.3)	**<0.001**
BRIEF‐A Metacognition Index *t* score	598	72.3 (17.5)	70.2 (18.1)	74.2 (17.0)	78.3 (19.9)	87.7 (16.3)	0.086
Functional Activities Questionnaire total score	597	5.7 (8.1)	4.3 (7.1)	6.5 (7.4)	13.7 (11.5)	23.2 (8.9)	**<0.001**
Geriatric Depression Scale‐15 total score	612	9.8 (4.4)	9.2 (4.9)	10.3 (4.3)	9.5 (4.5)	8.8 (4.1)	0.315
Beck Anxiety Inventory total score	317	17.1 (14.7)	13.1 (11.2)	17.2 (15.8)	12.6 (13.3)	12.4 (10.7)	0.340
Apathy Evaluation Scale total score	613	46.5 (13.3)	43.0 (14.4)	46.0 (14.1)	47.1 (13.6)	51.7 (13.0)	0.153
Barratt Impulsiveness Scale‐11	613	75.9 (16.8)	77.2 (16.6)	78.2 (16.0)	73.5 (16.9)	75.4 (14.3)	0.497
BRIEF‐A Behavioral Regulation Index *t* score	598	75.5 (16.2)	72.6 (17.1)	77.3 (16.7)	79.1 (18.8)	79.6 (16.4)	0.666
Dementia (y/n)	597	39 / 241 (16.2%)	11 / 87 (12.6%)	19 / 79 (24.1%)	70 / 133 (52.6%)	47 / 57 (82.5%)	**<0.001**

*Note*: This table presents the mean (standard deviation) scores of cognitive, functional, and behavioral assessments across different stages of CTE. Higher scores indicate greater severity or symptomatology on the respective scales. The table also reports dementia status (determined through the UNITE study) as a binary variable with counts and percentages. Sample sizes (*N*) vary by scale. *p* values are from analyses of covariance for continuous outcomes comparing across CTE stages (0, I, II, III, IV) and logistic regressions for binary outcomes (dementia). All analyses are adjusted for age at death. *p* values are based on main effects.

^a^ Mean (SD); *n*/*N* (%). If missingness, sample sizes may vary by CTE stage.

Abbreviations: BRIEF‐A, Behavior Rating Inventory of Executive Function‐Adult Version; CTE, chronic traumatic encephalopathy; SD, standard deviation.

**FIGURE 2 alz71032-fig-0002:**
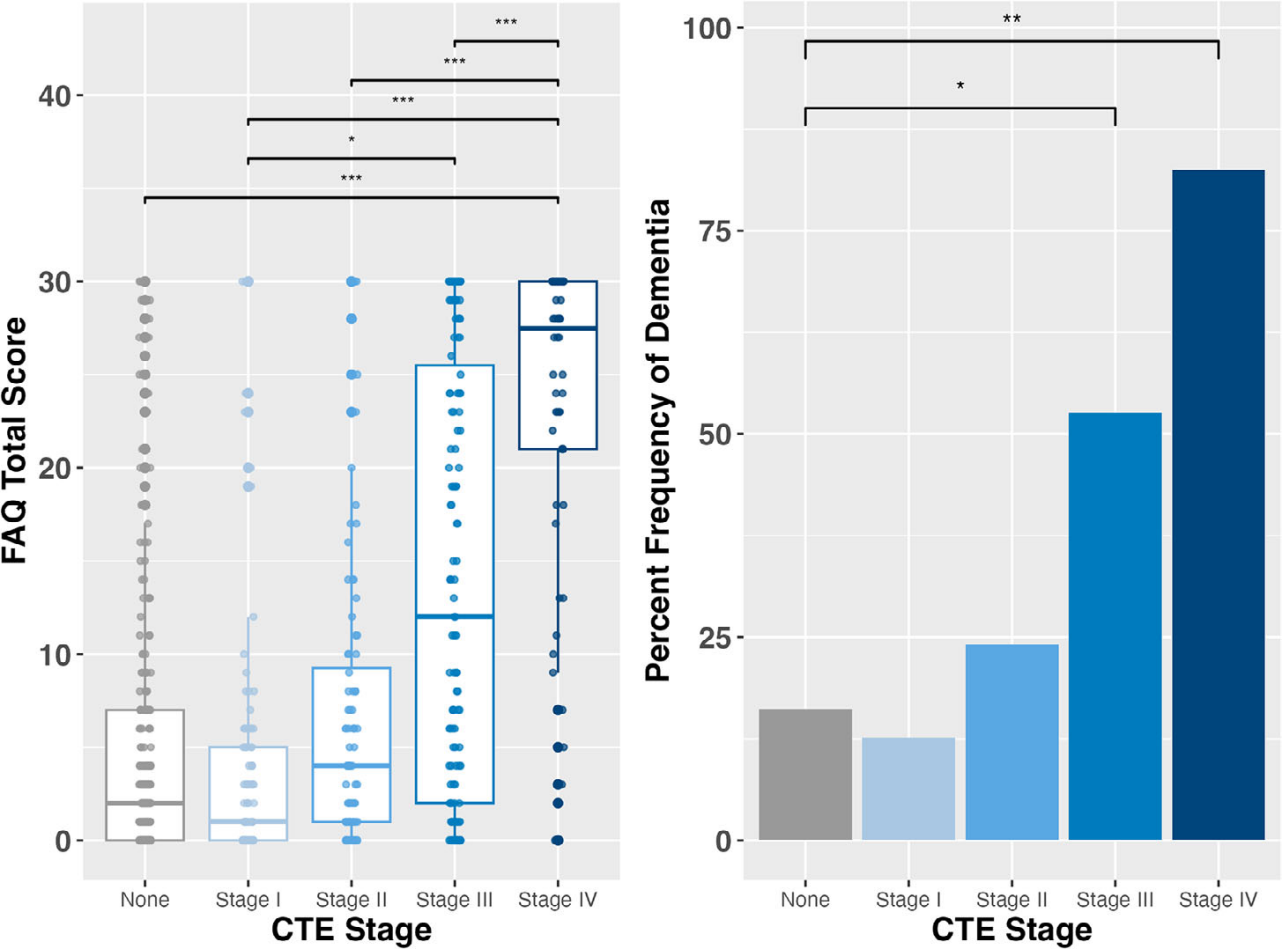
CTE stage and dementia status. CTE stage (0–IV) is associated with the FAQ total score and dementia status. Left panel, Boxplots illustrating the distribution of FAQ total scores across CTE stages (0–IV). The boxplots show the median (central line), interquartile range (box), and range excluding outliers (whiskers). Higher CTE stages are associated with increased FAQ total scores. Right panel, Bar graph showing the percent frequency of dementia diagnosis by CTE stage. Rates of dementia increase with higher CTE stage. Statistical significance is denoted as follows: *p < 0.05, **p < 0.01, ***p < 0.001. CTE, chromic traumatic encephalopathy; FAQ, Functional Activities Questionnaire

Higher CTE stage was associated with higher FAQ total score (Table 4 and Figure 2). Post hoc comparisons of estimated marginal means revealed that FAQ total scores were significantly higher in CTE stage IV compared to all other stages (No CTE: estimated marginal mean difference = 9.14, *p* < 0.001; stage I: estimated marginal mean difference = 10.08, *p* < 0.001; stage II: estimated marginal mean difference = 9.40, *p* < 0.001; stage III: estimated marginal mean difference = 6.76, *p* < 0.001). A significant difference was observed between CTE stage I and stage III (estimated marginal mean difference = 3.33, *p* = 0.026), with stage III having higher FAQ scores than stage I. No other significant differences were found. Results remained when the model was repeated, adjusting for white matter rarefaction and cerebrovascular pathologies and treatment for substance use. When the model was repeated including Braak stage, all post hoc comparisons remained with an additional difference between CTE stage III and no CTE (estimated marginal mean difference = 3.20, *p* = 0.036), with stage III having higher FAQ scores than no CTE. When the model was repeated with hippocampal sclerosis as a covariate, all post hoc comparisons remained similar.

### 3.3 CTE stage and clinical scales

**FIGURE 3 alz71032-fig-0003:**
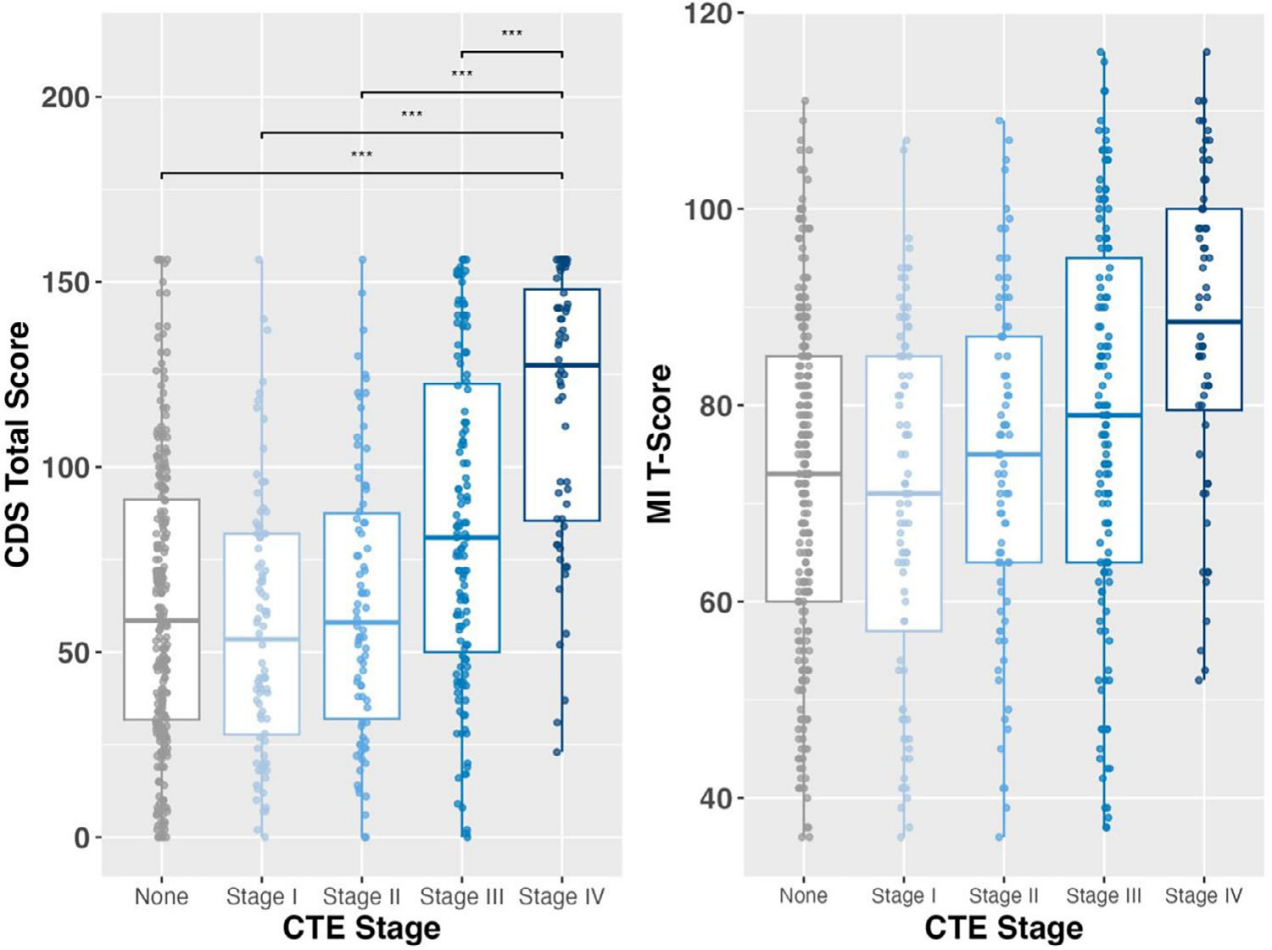
CTE stage and informant‐reported cognitive symptoms. Boxplots illustrate the relationship between CTE stage (0–IV) and informant‐reported measures of cognitive function, including the CDS (left panel) and the BRIEF‐A MI (right panel). Each boxplot displays the median (central line), interquartile range (box), and whiskers representing data variability excluding outliers. Higher CTE stages are associated with increased CDS scores, indicating greater cognitive symptoms. Statistical significance is denoted by asterisks: *p < 0.05, **p < 0.01, ***p < 0.001. BRIEF‐A MI, Behavior Rating Inventory of Executive Function‐Adult Version Metacognition Index; CDS, Cognitive Difficulties Scale; CTE, chronic traumatic encephalopathy

Higher CTE stage was associated with greater informant‐reported cognitive symptoms on the CDS (*p* = 0.001) but not the BRIEF‐A MI (*p* = 0.086; Table 4 and Figure 3). Post hoc comparisons of estimated marginal means revealed that the CDS total scores were significantly higher in CTE stage IV compared to all other stages (No CTE: estimated marginal mean difference = 30.03, *p* < 0.001; stage I: estimated marginal mean difference = 34.16, *p* < 0.001; stage II: estimated marginal mean difference = 32.61, *p* < 0.001; Stage III: estimated marginal mean difference = 23.94, *p* < 0.001). Those with CTE stage IV had CDS scores that were nearly twice as high (on average) as those without CTE. Significant differences remained when the model was repeated adjusting for white matter rarefaction and cerebrovascular pathologies and treatment for substance use. All results remained when models were repeated including Braak stage and hippocampal sclerosis.

### 3.4 CTE stage and mood and behavioral scales

**FIGURE 4 alz71032-fig-0004:**
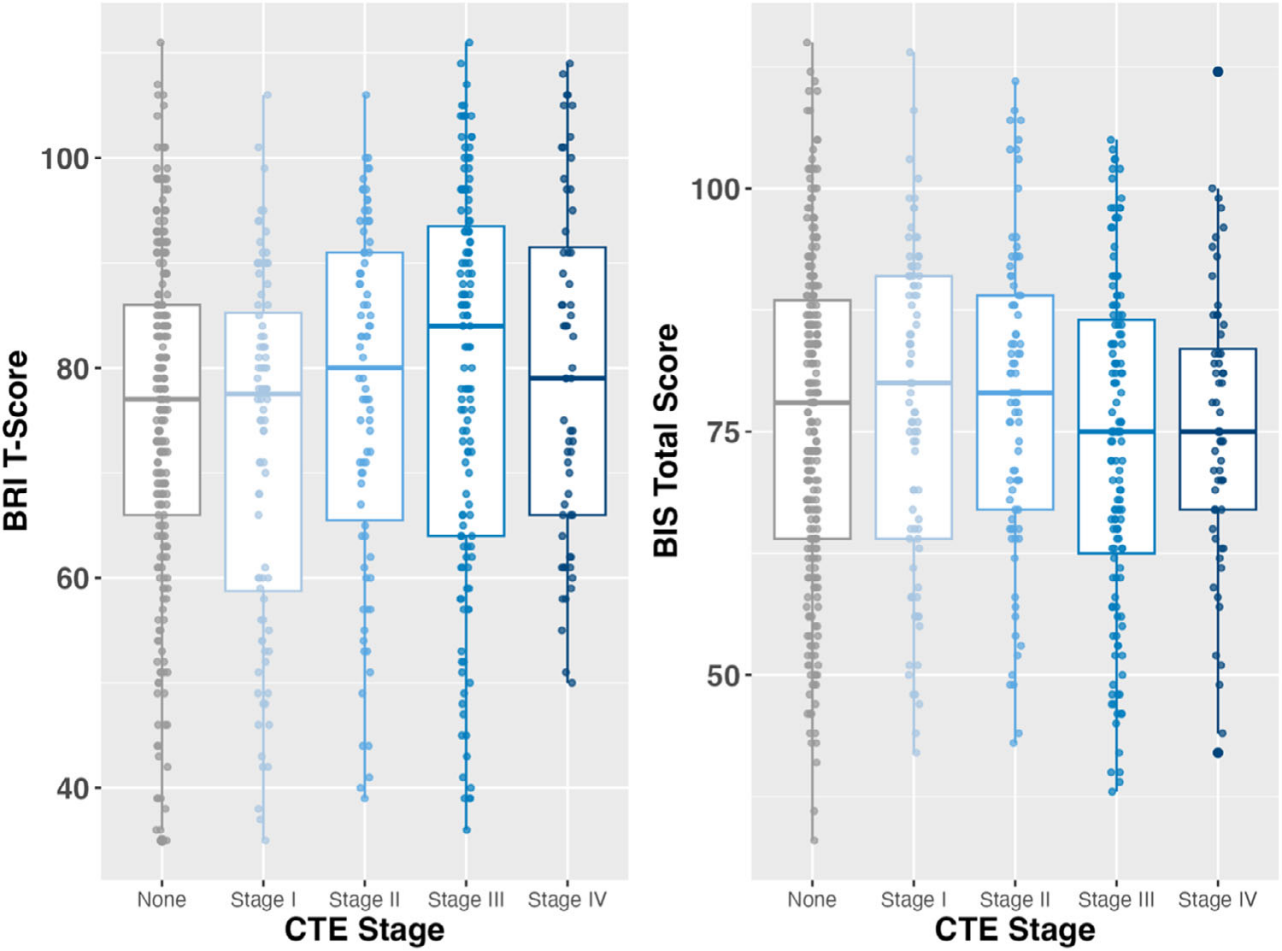
CTE stage and informant‐reported behavioral symptoms. Boxplots depict the relationship between CTE stage (0–IV) and informant‐reported behavioral symptom measures, including the BRIEF‐A BRI (left panel) and BIS‐11 (right panel). Each boxplot displays the median (central line), interquartile range (box), and whiskers representing the data range excluding outliers. No significant differences were observed across CTE stages. BIS‐11, Barratt Impulsiveness Scale‐11; BRIEF‐A BRI, Behavior Rating Inventory of Executive Function‐Adult Version Behavioral Regulation Index; CDS, Cognitive Difficulties Scale; CTE, chromic traumatic encephalopathy

CTE stage of any severity was not associated with any of the mood scales, including the GDS‐15, BAI, or AES. There was no relationship between CTE stage of any severity and either of the behavioral scales, including the BIS‐11 and BRIEF‐A BRI (Figure 4).

### 3.5 CTE stage high versus low

We examined CTE categorized as low (stages I and II) and high (stages III and IV) while accounting for age at death. High‐stage CTE was associated with increased odds of having dementia (OR = 2.53, 95% CI = 1.473–4.371, *p* = 0.001). High‐stage CTE was also associated with higher CDS (*p* = 0.001) and FAQ (*p* < 0.001) scores compared to no CTE. It was not associated with BRIEF‐A MI scores (*p* = 0.427). Low‐stage CTE was not associated with dementia status or any of the scales.

### 3.6 Age stratification

Sample characteristics stratified by median age 52 are included in Table S1 in supporting information. Of the sample, 313 were 52 or older (*n* = 87 no CTE, *n* = 58 low CTE, *n* = 168 high CTE). Of those who were 52 or older, there continued to be age differences by CTE stage, but less so compared to the entire sample. When accounting for age of death, CTE stage IV was associated with 4.46 (95% CI = 1.90–11.23, *p* = 0.001) increased odds of having dementia compared to no CTE. CTE stages I, II, and III were not associated with increased odds of having dementia when compared to no CTE. Higher CTE stage was also associated with higher FAQ and CDS scores.

High‐stage CTE was associated with increased odds of having dementia among those 52 years or older (OR = 2.32, 95% CI = 1.28–4.25, *p* < 0.001). Low‐stage CTE was not associated with dementia status. High‐stage CTE was associated with higher CDS and FAQ scores. Results are summarized in Tables S2 and S3 in supporting information.

## 4 DISCUSSION

The objective of this study was to investigate the association between CTE neuropathology and informant‐reported cognitive, functional, mood, and behavioral symptoms among 614 brain donors from the UNITE brain bank. The sample did not have AD, LBD, FTLD, or MND neuropathological diagnoses to determine the independent effect of CTE on symptoms. CTE stages III and IV were associated with worse cognitive and functional symptoms, including a more than four times (stage IV) increased odds for dementia prior to death. Effects were independent of age. These associations remained even after adjusting for white matter rarefaction, arteriolosclerosis, atherosclerosis, Braak stage, hippocampal sclerosis, and history of substance use treatment. Stages I and II were[Fig alz71032-fig-0002], [Fig alz71032-fig-0003], [Fig alz71032-fig-0004] not associated with cognitive or functional symptoms (reference group: no CTE). There were no statistically significant associations between CTE neuropathology (0–IV) and informant‐reported mood or behavioral symptoms. These findings suggest that high‐stage CTE neuropathology in and of itself contributes to cognitive and functional symptoms, while the contribution of low‐stage CTE neuropathology to symptoms remains uncertain.

High CTE stage was associated with worse cognitive symptoms and increased odds for *ante mortem* dementia in this sample of brain donors, independent of other neurodegenerative and non‐neurodegenerative neuropathologies. CTE stage III and IV were associated with 2.12 and 4.48 odds, respectively, for having *ante mortem* dementia. Research has shown an OR of 4.15 between AD neuropathology (NIA‐Reagan) and AD dementia.^25^ TES is the proposed clinical syndrome of CTE, and the criteria require the presence of progressively worsening cognitive impairment (i.e., in episodic memory or executive function) and/or neurobehavioral dysregulation.^7^ However, the specificity of the TES criteria and their associated symptoms to CTE has been debated. Iverson et al. argued that CTE may not be progressive, and the described symptoms are common in the general population and thus not associated with CTE neuropathology.^26^ A recent review and meta‐analysis of cognitive impairment and dementia in autopsy‐confirmed CTE concluded a lack of certainty on the association between CTE neuropathology and symptoms due to the presence of co‐morbid neuropathologies based on a small sample.^10^ The presence of co‐morbid neuropathologies in neurodegenerative disease is often viewed as the “rule and not the exception.” In our study, 65% of the CTE cases had no AD, LBD, FTLD, or MND, likely because of the young age of death relative to other neurodegenerative disease brain banks, particularly in those who had low‐stage CTE. When present, co‐morbid neuropathology does not negate the contribution of CTE to symptoms; rather, the percent contribution to the symptoms might vary by the pathology.^9^, ^25^ Showing associations between the neuropathology and symptoms is important for understanding the clinical meaning of the pathology and informing clinical diagnostic criteria. This study provides evidence that CTE p‐tau alone precipitates cognitive symptoms, supporting their continued inclusion in future iterations of the TES criteria.

AD can be co‐morbid with CTE, and the two diseases can have similar presentations. The neuropsychological profile of former elite football players has been characterized to predominantly include deficits in episodic memory—similar to AD.^27^ Of the brain donors who had stage III and IV CTE and no AD in the sample, AD was the most common diagnosis made during life. Misdiagnosis of CTE as AD is likely to be common given the similar presentations when dementia is present, and it is important for clinicians to appropriately use currently available biomarkers to rule out AD in cases who have extensive head trauma exposure. Continued research that clarifies the neuropsychological distinctions and patterns between CTE and AD is also needed.

Unlike cognitive symptoms, we did not observe an association between CTE stage and mood and behavioral symptoms, including neurobehavioral dysregulation. These symptoms begin at a young age and are common to people without CTE.^2^, ^17^ Our null findings are consistent with our previous research showing no or weak associations between CTE p‐tau neuropathology and mood and behavior symptoms.^16^ It is still possible that CTE p‐tau contributes to these symptoms and that limitations of our autopsy studies (e.g., retrospective interviews, measurement) account for the absence of findings. Alternatively, these mood and behavioral symptoms might be better accounted for by other factors. There could be non‐tau pathological pathways that might account for the neurobehavioral dysregulation (e.g., white matter rarefaction, arteriolosclerosis).^11^ The cross‐sectional design might contribute to the null findings. A study of AD pathology of the locus coeruleus found that symptoms of depression, anxiety, agitation, and sleep disturbances are elevated in Braak stages I and II before regressing as pathology progresses.^28^ Neurobehavioral dysregulation should be closely scrutinized in future revisions of the TES criteria.

No associations between low‐stage CTE (I/II) and informant‐reported cognitive and functional symptoms, nor dementia were found. The minimum threshold by which CTE confers symptoms is poorly understood, with questions raised about whether CTE stages I and II are sufficient pathology to clinically manifest. This is not unexpected in the context of literature on other neurodegenerative diseases. Low stages of AD (as estimated by tau positron emission tomography [PET]) also do not generally correspond to cognitive symptoms,^29^ whereas Braak stages of V and VI (as estimated by tau PET) demonstrate the strongest concordance with dementia.^29^, ^30^ Stage I and II CTE are generally characterized by patchy and low‐density pathognomonic lesions of CTE, typically in the frontal and temporal cortices.^5^, ^6^ Recent work from the UNITE brain bank among young brain donors who nearly all had stage I and II CTE did not observe an association between CTE and cognitive, mood, or behavioral symptoms. CTE stage I and II could represent a pre‐clinical phase of the disease, similar to AD, in which the neuritic amyloid plaques begin ≥ 15 years prior to the onset of symptoms.^31^ It may be more nuanced in CTE as many of the individuals could have symptoms related to pathologies from RHI, making it difficult to classify them as “pre‐clinical.” Nevertheless, the current findings provide evidence that CTE‐specific symptoms might not appear until stage III. It remains important to detect CTE stage I and II in vivo, as this might represent the opportune time for therapeutic intervention. Our certainty that CTE is present and contributing to symptoms is likely to be strongest at stages III or IV.

There are several limitations to the findings of this study. While exclusions of neuropathologies were not exhaustive, we accounted for common symptom driving diagnostic neuropathologies, and it would be challenging to fully isolate CTE from all potential neuropathologies. We did not exclude hippocampal TDP‐43 inclusions because nearly all brain donors with these inclusions had high‐stage CTE (and dementia). Previous work has discussed the interplay among RHI, CTE, and TDP‐43.^23^ There were age differences across the CTE stages in the entire sample. We statistically controlled for age and conducted age‐stratified models in which age differences were attenuated, and effects remained similar. Age might still represent an important confounder. All brain donors had RHI, and neuronal loss and neuroinflammation can begin at young ages, perhaps before the emergence of CTE tau pathology.^18^ Therefore, age‐matched individuals without RHI are needed to fully understand the contribution of CTE stage, particularly the low stages, to symptoms.

The accuracy of the symptoms reported by next‐of‐kin could be affected by recall biases. Prospective studies that monitor individuals serially during life with objective neurobehavioral measures and then validate them against CTE neuropathology *post mortem* are needed to confirm our findings. These types of clinical–pathological validation studies will take many years. In the interim, development and validation of an in vivo biomarker for CTE neuropathology would allow for the in‐life investigation of the associations between symptoms (and their progression) across the different CTE stages. Another limitation of the study is selection bias, as these donors are more likely to be symptomatic and have CTE neuropathology. It is possible that the associations between CTE stage and symptoms were overestimated. The generalizability of the findings is limited due to selection biases, as well as the sample predominantly consisting of white males who played American football, limiting our understanding of the associations in females, underrepresented groups, and other sources of RHI. CTE stage is also based on the distribution of p‐tau in the brain, rather than the density of p‐tau pathology. It could be that the overall amount of p‐tau in certain regions is more associated with the clinical manifestation of CTE than the spread of p‐tau pathology.

## 5 CONCLUSIONS

This study found an association between high CTE stage (III/IV) and increased odds for having cognitive and functional symptoms, in the absence of co‐morbid neurodegenerative disease pathologies. Low CTE stage (I/II) was not associated with cognitive and functional symptoms, and CTE neuropathology was not associated with mood and behavioral symptoms. This study is among the first to provide evidence for advanced CTE neuropathology alone as being associated with dementia. Our findings suggest that low‐stage CTE might not be sufficient to manifest clinically. The etiology of mood and behavioral symptoms often observed in this setting might not be fully explained by CTE. Prospective clinical–pathological studies are needed to validate the associations between CTE neuropathology and objectively measured symptom phenotypes.

## CONFLICT OF INTEREST STATEMENT

Dr. Nowinski is the cofounder and chief executive officer of the Concussion and CTE Foundation; reported non‐financial support (travel reimbursement) from the NFL Players Association as a member of the Mackey‐White Health & Safety Committee, WWE, AEW (All Elite Wrestling), World Rugby, and TNA; and serves as an advisor and options holder for Oxeia Biopharmaceuticals, PreCon Health, and StataDx outside the submitted work. Dr. Katz reports royalities from Springer/Demos publishers for a text book on Brain INjury Medicine and honorarium and travel support (reimbursement) from Cleveland Clinic for a presentation on CTE. Dr. Cantu reports royalties from Houghton Mifflin Harcourt; compensation for expert legal opinion to the National Collegiate Athletic Association and National Hockey League; consults for the Concussion and CTE Foundation; is senior advisor and paid consultant to the NFL Head Neck & Spine Committee; is a member of the Mackey‐White Committee of the National Football League Players Association; is vice president of National Operating Committee on Standards for Athletic Equipment and chair scientific advisory committee and cofounder of Medical Director Concussion and CTE Foundation; and is on the Medical Science Committee for the National Collegiate Athletic Association Student‐Athlete Concussion Injury Litigation. Dr. Stern reports personal fees from Biogen and Lundbeck outside the submitted work; is a member of the Mackey‐White Committee of the National Football League Players Association; and receives royalties for published neuropsychological tests from Psychological Assessment Resources Inc. He reports honoraria for invited lectures. Dr. Stein reports support (travel reimbursement) from the Concussion and CTE Foundation. Dr. McKee is a member of the Mackey‐White Committee of the National Football League Players Association and reports other funding from the National Institutes of Health, Department of Veteran Affairs, the Buoniconti Foundation and the MacParkman Foundation during the conduct of the study. She reports honorarium for speaking engagements. Dr. Daneshvar serves as an expert witness in legal cases involving brain injury and concussion; receives funds from the Football Players Health Study at Harvard University, which is funded by the National Football League Players Association (NFLPA); evaluates patients for the MGH Brain and Body TRUST Center, sponsored in part by the NFLPA; and serves as an advisor and options holder for StataDx outside the submitted work. Dr. Mez reports honorarium for invited presentations. Dr. Alosco receives royalties from Oxford University Press Inc for a textbook outside the submitted work. He receives honorarium from the Michael J. Fox Foundation Inc for services unrelated to this study, and he has received honorarium for CME and other invited presentations on CTE. He receives research support from Life Molecular Imaging Inc and Department of Defense. No other disclosures were reported. Author disclosures are available in the supporting information.

## CONSENT STATEMENT

Next of kin provided consent for brain donation.

## Supporting information



Supporting Information

Supporting Information
