# Supplementary material for: CTE neuropathology alone is associated with dementia and cognitive symptoms
Source: Alzheimers Dement. 2026 Jan 27;22(1):e71032. doi: 10.1002/alz.71032 (PMC12836376; doi:10.1002/alz.71032)
Supplement: Supplementary file 1 — Supporting Information [file ALZ-22-e71032-s001.docx]

**Supplementary Materials**

**Supplemental Table 1: Sample Characteristics by CTE Stage in donors 52 years or older at death**

|  | **N** | **None**  N = 87^1^ | **Low**  N = 58^1^ | **High**  N = 168^1^ | **p-value**^2^ |
| --- | --- | --- | --- | --- | --- |
| Age of death | 313 | 66 (10) | 65 (9) | 72 (11) | <0.001 |
| Sex (% Male) | 313 | 83 (95%) | 58 (100%) | 168 (100%) | 0.007 |
| Race | 312 |  |  |  | <0.001 |
| White |  | 85 (98%) | 48 (83%) | 129 (77%) |  |
| Black |  | 2 (2.3%) | 9 (16%) | 37 (22%) |  |
| Other |  | 0 (0%) | 1 (1.7%) | 1 (0.6%) |  |
| Education Level | 313 |  |  |  | 0.003 |
| Some High School or less |  | 0 (0%) | 1 (1.7%) | 0 (0%) |  |
| High School Diploma/GED |  | 9 (10%) | 0 (0%) | 4 (2.4%) |  |
| Some College |  | 18 (21%) | 14 (24%) | 24 (14%) |  |
| College Degree or higher |  | 60 (69%) | 43 (74%) | 140 (83%) |  |
| Years of Football Play | 267 | 8.2 (4.6) | 12.9 (5.0) | 15.3 (5.6) | <0.001 |
| Highest level of football play | 267 |  |  |  | <0.001 |
| Pre High School |  | 3 (5.6%) | 0 (0%) | 0 (0%) |  |
| High School |  | 20 (37%) | 3 (5.8%) | 6 (3.7%) |  |
| College |  | 22 (41%) | 20 (38%) | 44 (27%) |  |
| Semi-Professional |  | 1 (1.9%) | 6 (12%) | 5 (3.1%) |  |
| Professional |  | 8 (15%) | 23 (44%) | 106 (66%) |  |
| Position played at highest level | 257 |  |  |  | <0.001 |
| Offensive Line |  | 7 (14%) | 14 (29%) | 30 (19%) |  |
| Tight End |  | 0 (0%) | 1 (2.1%) | 7 (4.4%) |  |
| Quarterback |  | 5 (10%) | 2 (4.2%) | 5 (3.1%) |  |
| Running Back |  | 7 (14%) | 2 (4.2%) | 28 (18%) |  |
| Wide Receiver |  | 0 (0%) | 2 (4.2%) | 5 (3.1%) |  |
| Defensive Line |  | 6 (12%) | 13 (27%) | 22 (14%) |  |
| Linebacker |  | 4 (8.2%) | 7 (15%) | 29 (18%) |  |
| Defensive Back |  | 2 (4.1%) | 2 (4.2%) | 19 (12%) |  |
| Other/Special Teams |  | 4 (8.2%) | 2 (4.2%) | 0 (0%) |  |
| Multiple |  | 14 (29%) | 3 (6.3%) | 15 (9.4%) |  |
| Played other contact sports | 313 | 33 (38%) | 17 (29%) | 46 (27%) | 0.216 |
| Treated for drugs or alcohol | 288 | 19 (24%) | 19 (36%) | 34 (22%) | 0.122 |
| Cause of Death | 310 |  |  |  | <0.001 |
| Suicide |  | 18 (21%) | 7 (12%) | 7 (4.2%) |  |
| Accidental Overdose |  | 3 (3.5%) | 4 (7.0%) | 2 (1.2%) |  |
| Cardiovascular Disease |  | 23 (27%) | 20 (35%) | 34 (20%) |  |
| Neurodegenerative |  | 8 (9.3%) | 4 (7.0%) | 57 (34%) |  |
| Cancer |  | 8 (9.3%) | 6 (11%) | 21 (13%) |  |
| Other |  | 23 (27%) | 15 (26%) | 45 (27%) |  |
| Injury |  | 3 (3.5%) | 1 (1.8%) | 1 (0.6%) |  |
| ^1^Mean (SD); n (%) | | | | | |
| ^2^Kruskal-Wallis rank sum test; Fisher's exact test; Pearson's Chi-squared test | | | | | |

**Supplemental Table 2:** Cognitive, Functional, and Neuropsychiatric Scale Summary Statistics by CTE Stage among donors **52 years or older** at death

| **Characteristic** | **N** | **CTE**  **Stage 0**  N = 87^1^ | **CTE**  **Stage I**  N = 25^1^ | **CTE**  **Stage II**  N = 33^1^ | **CTE**  **Stage III**  N = 109^1^ | **CTE**  **Stage IV**  N = 59^1^ | | **p-value** |
| --- | --- | --- | --- | --- | --- | --- | --- | --- |
| Cognitive Difficulties Scale Total Score | 313 | 75.0 (40.7) | 69.1 (34.4) | 79.3 (35.3) | 90.2 (43.0) | 116.8 (37.5) | | **<0.001** |
| Meta-cognition Index T-Score | 313 | 75.8 (19.1) | 74.1 (19.4) | 79.9 (15.7) | 80.8 (19.3) | 87.9 (16.3) | | 0.060 |
| Functional Activities Questionnaire Total Score | 306 | 10.6 (10.4) | 9.3 (10.1) | 10.6 (9.3) | 16.1 (11.4) | 23.5 (8.6) | | **<0.001** |
| Geriatric Depression Scale Total Score | 313 | 10.0 (4.4) | 8.9 (4.6) | 10.7 (3.9) | 9.4 (4.5) | 8.7 (4.1) | | 0.523 |
| Beck Anxiety Inventory Total Score | 159 | 15.1 (15.3) | 9.7 (12.1) | 10.3 (15.0) | 13.4 (14.1) | 11.9 (10.4) | | 0.647 |
| Apathy Evaluation Scale Total Score | 313 | 47.2 (14.0) | 44.8 (11.9) | 47.4 (13.1) | 48.3 (13.0) | 51.7 (13.1) | | 0.284 |
| Barratt Impulsiveness Scale T-Score | 313 | 71.6 (15.6) | 71.0 (15.1) | 79.1 (14.7) | 72.9 (16.2) | 75.1 (14.3) | | **0.030** |
| Behavioral Regulation Index T-Score | 313 | 79.1 (15.6) | 75.4 (17.7) | 83.4 (15.3) | 79.6 (19.2) | 79.9 (16.4) | | 0.536 |
| Dementia (y/n) | 305 | 33/85 (38.8%) | 8/24 (33.3%) | 17/33 (51.5%) | 64/107(59.8%) | 47/56 (83.9%) | | **<0.001** |
| ^1^Mean (SD); n / N (%) | | | | | | |  | |
| This table presents the mean (standard deviation) scores of cognitive, functional, and behavioral assessments across different stages of CTE (0-4) among brain donors 52 years or older. Higher scores indicate greater severity or symptomatology on the respective scales. The table also reports the history of dementia as a binary variable with counts and percentages. Sample sizes (N) vary by scale. p-values are from analysis of covariance (ANCOVA) for continuous outcomes comparing across CTE stages (0, I, II, III, IV) and logistic regressions for binary outcomes (dementia). All analyses are adjusted for age at death. P-values are based on main effects. While these was a main effect for the Barratt Impulsiveness Scale, post-hoc tests showed no differences by stage. | | | | | | | | |

**Supplemental Table 3:** Cognitive, Functional, and Neuropsychiatric Scale Summary Statistics by CTE Stage among donors **52 years or older** at death

| **Characteristic** | **N** | **None**  N = 87^1^ | **Low**  N = 58^1^ | **High**  N = 168^1^ | **p-value** |
| --- | --- | --- | --- | --- | --- |
| Cognitive Difficulties Scale Total Score | 313 | 75.0 (40.7) | 74.9 (35.0) | 99.5 (43.0) | **0.001** |
| Meta-cognition Index T-Score | 313 | 75.8 (19.1) | 77.4 (17.4) | 83.3 (18.6) | 0.100 |
| Functional Activities Questionnaire Total Score | 306 | 10.6 (10.4) | 10.0 (9.6) | 18.7 (11.1) | **<0.001** |
| Geriatric Depression Scale Total Score | 313 | 10.0 (4.4) | 9.9 (4.3) | 9.1 (4.4) | 0.888 |
| Beck Anxiety Inventory Total Score | 159 | 15.1 (15.3) | 10.0 (13.6) | 12.7 (12.6) | 0.332 |
| Apathy Evaluation Scale Total Score | 313 | 47.2 (14.0) | 46.2 (12.6) | 49.5 (13.1) | 0.303 |
| Barratt Impulsiveness Scale T-Score | 313 | 71.6 (15.6) | 75.6 (15.3) | 73.6 (15.6) | 0.156 |
| Behavioral Regulation Index T-Score | 313 | 79.1 (15.6) | 79.9 (16.7) | 79.8 (18.2) | 0.945 |
| Dementia (y/n) | 305 | 33 / 85 (38.8%) | 25 / 57 (43.9%) | 111 / 163 (68.1%) | **<0.001** |
| ^1^Mean (SD); n / N (%) | | | | |  |
| This table presents the mean (standard deviation) scores of cognitive, functional, and behavioral assessments across different stages of CTE (none, low, high) among brain donors 52 years or older. Higher scores indicate greater severity or symptomatology on the respective scales. The table also reports the history of dementia as a binary variable with counts and percentages. Sample sizes (N) vary by scale. p-values are from analysis of covariance (ANCOVA) for continuous outcomes comparing across CTE stages (0, I, II, III, IV) and logistic regressions for binary outcomes (dementia). All analyses are adjusted for age at death. | | | | | |
